# The Relationship between Performance, Body Composition, and Processing Yield in Broilers: A Systematic Review and Meta-Regression

**DOI:** 10.3390/ani12192706

**Published:** 2022-10-08

**Authors:** Diego A. Martinez, Jordan T. Weil, Nawin Suesuttajit, Cole Umberson, Abdullah Scott, Craig N. Coon

**Affiliations:** Department of Poultry Science, University of Arkansas, Fayetteville, AR 72701, USA

**Keywords:** body composition, body protein, body fat, lean mass, breast meat, net energy, modeling, meta-regression, meta-analysis, broilers

## Abstract

**Simple Summary:**

Performance, body composition, and processing yield are important traits in broiler production. Quantifying their association will favor making decisions and better understanding the underlying processes involved in productive and economic efficiency. This study aimed to approach this topic by modeling the relationships between these groups of variables by applying regression to data from multiple studies. The study suggests that regardless of the intervention applied and the type of broilers, the feed conversion ratio improves when the body weight gain also increases. The study’s results support the use of body composition to predict the carcass conformation of broilers and their economic value. The information provided herein may particularly be of value to point out in the right direction further modeling efforts in areas related to poultry economics and sustainability.

**Abstract:**

This study aims to model the relationship among performance, whole body composition, and processing yield through meta-regression. Scientific papers found in Scopus and Google Scholar were included if they reported results and variability values of an actual experiment in the three mentioned groups of variables using a single broiler genetic line. Weighted mean effect sizes were determined with a random model, the risk of bias was determined, and heterogeneity was considered an indicator of usefulness. Meta-regressions considered the effect sizes of the response variable and the percent change in one or more variables as predictors. A 78-row database was built from 14 papers, including nine factors tested on 22,256 broilers. No influencing bias was found, and the data was determined useful. Meta-regressions showed that the changes in body weight gain (BWG) are inversely related to the effects in feed conversion ratio (FCR) (*p* < 0.001) and that the changes in FCR and effects in protein-to-fat gain (PFG) are directly related (*p* < 0.001). The changes in PFG and the effects on carcass conformation or the market value of birds are directly related (*p* < 0.001). In conclusion, body composition predicts carcass conformation and its market value, supporting its use to predict the economic value of broilers.

## 1. Introduction

Modern broilers show increasing growth rates, improved feed conversions, and increased breast meat size and yield, which play a pivotal role in the economics and sustainability of poultry production. Indeed, across the years, broilers have become leaner [1], and the industry aims to increase breast meat production due to its higher market price.

To achieve this aim, operation professionals need to be able to determine, ahead of time, what the final results will be, particularly the body weight (BW) and the breast yield of the flocks. However, appropriate indicators are needed, and it is necessary to more accurately determine how likely it is for the performance to correlate to processing traits. Similarly, a correct cost-benefit balance between lowering the feed conversion ratio (FCR) and increasing breast meat production is considered by industry nutritionists to optimize the feeding cost and increase the market value of the broilers, respectively. To do so, dealing with the relationship between performance and processing yield traits is crucial.

Optimum production requires the best possible diet formulation strategies, so the industry will eventually move towards a productive energy system to more accurately take into account the effective use of nutrients to produce meat [2]. The Arkansas Net Energy system [3] has been developed by taking into account the body energy gain (net energy for gain) based on the protein and fat gains (determined by Dual Energy X-Ray Absorptiometry) multiplied by their corresponding caloric coefficients, and the fasting heat production (net energy for maintenance; determined with indirect calorimetry). This system has been shown to be well correlated with the performance of broilers [4], and accurate equations have been validated to predict the fasting heat production based on the body composition [5], thus reducing the dependence on respiratory chambers. However, a better understanding of the relationship between FCR, the dynamics of body composition, and breast meat production will support future modeling approaches to predict body composition and ease the determination of body energy gain. A better understanding of broiler performance and breast meat production will also support the development of strategies to attenuate meat quality issues [6].

Consequently, better quantifying the association between performance, body composition, and processing yield will support production professionals, nutritionists, and researchers to make decisions and better understand the underlying processes involved in productive and economic efficiency. Most studies on the relationships between these variables have focused on comparing genetic lines [7] or determining individual variations within the same genetic line [8,9]. Indeed, negative phenotypic and genotypic correlations have been reported between FCR and body weight gain (BWG) when the intra-genetic line variability was explored in individual birds [10,11]. Furthermore, it has been reported that a positive linear relationship exists between percent increases in the body protein content (g) and in the breast meat or thigh weights (g) as the bird grows [12]. However, as can be ascertained from the existing literature, no study has determined how the changes in one of these response variables as the result of applying multiple interventions correlate with changes in another. Modeling these relationships by applying a meta-regression multifactorial approach including studies that assessed diverse factors, may provide a broader point of view, clearer insight, and robust inferences. The present study aims to summarize broiler studies reported in the literature whose response variables included performance, body composition, and processing yield, regardless of the factor studied, to model the relationships between specific variables through meta-regression.

## 2. Materials and Methods

The meta-analysis was conducted and reported following the Preferred Reporting Items for Systematic Reviews and Meta-analysis (PRISMA) 2020 guidelines [13].

### 2.1. General Approach

A multifactorial approach [14,15] was applied to favor generalization, so the data used to build the models was not limited to a single factor tested but to any whose effects were measured in productive performance, body composition gain, and processing yield.

### 2.2. Eligibility Criteria

The scientific papers used fitted the following inclusion criteria: An study was included if it (1) was an actual experiment, (2) reported results in the three groups of variables (performance, body composition, processing yield) as the result of testing any experimental treatment using a single broiler genetic line, (3) the sample analyzed (i.e., to determine body composition) was the whole body (including feathers), and (4) reported a variability index per response variable.

### 2.3. Information Sources, Search Strategy, and Selection Process

Scientific papers were searched in Scopus [16] and Google Scholar [17] to identify papers published until December 2021 considering the following keywords in English: “{*breast yield*} AND *broilers* AND {*body protein*} OR {*protein deposition*} OR {*protein gain*}”. The following keywords in Portuguese were also used: “*peito* AND *frango* AND {*proteína corporal*} OR {*deposição protéica*} OR {*ganho de proteína*}”. Only articles in English and Portuguese were included. The documents gathered were examined. Those not related to the focus of the present review, thesis or dissertations, documents that were not papers published in peer-reviewed scientific journals, and duplicates were removed. One reviewer (D.A.M.) selected the papers by inspecting the titles, abstracts, and the whole body of the manuscripts and considering the criteria mentioned earlier. The reduced set of papers was approved by a second reviewer (C.N.C.).

### 2.4. Data Collection Process, Data Items, and Effect Measures

A database was built where each row consisted of a set of independent data and included all the data corresponding to a comparison of two treatments from the same experiment at the same age, obtaining as many datasets as non-redundant comparisons were observed. Data from different experimental periods within a paper were considered completely independent and conformed separate rows in the database [14]. Every row included identifying information about the experiment (reference, journal, year) and the treatments compared, reported treatment values in each response variable, number of replications, and variability values. Column sections included BWG (g/bird/d), FCR (accounted as one point for every g/g value [18]), body protein gain ratio (PGR; g/bird/d), body fat gain ratio (FGR; g/bird/d), protein-to-fat gain ratio (PFG), carcass yield (CAR; %), breast- (*Pectoralis major* + *P. minor*) to-legs (leg quarters) ratio (BLR), and market value (MKV; USD/bird; defined as the value of the whole breast meat plus the leg quarters value). One reviewer (D.A.M.) collected the data from each paper. For every response variable in each data row, the effect size was considered the difference between the means of the two treatments being compared [19]. Possible outlying values were not assessed or removed.

### 2.5. Synthesis Methods

Two synthesis stages applied in the study consisted of (A) grouping the effect sizes by response variable to determine their heterogeneity and (B) including the percent (%) changes of other response variables considered as moderators to determine the relationship between the changes in the predictor (i.e., moderator) and response variables. All studies providing data were considered eligible for each synthesis.

Some response variables were calculated based on the data reported in each study. The following calculations were applied:

(1) PFG=PGR/FGR,

where PFG, PGR, and FGR were all expressed in g/bird/d,

(2) BLR=B/L,

where BLR was the breast-to-legs ratio, and B and L were the breast and leg quarters, respectively (weight or yield; both in the same unit), and

(3) $$\mathrm{MKV}=\left(\mathrm{BRE}\times {\mathrm{BRE}}_{P}+\mathrm{LQU}\times {\mathrm{LQU}}_{P}\right)\times \mathrm{BW},$$

were BRE was the breast meat yield (%), LQU was the leg quarters yield (%), ${\mathrm{BRE}}_{P}$ and ${\mathrm{LQU}}_{P}$ were the market prices ($/g) of the whole deboned breast (without wings) and leg quarters, respectively [20], and BW was expressed in g. If PGR was not reported but nitrogen retention, then the PGR was calculated as follows,

(4) PGR=NR×6.25,

where PGR was expressed in g/bird/g and NR was the nitrogen retention (g/bird/d). If FGR was not reported, but energy gain was available, FGR was calculated based on the caloric coefficients of body protein and fat by rearranging an equation previously reported [21] to clear the body fat content, as follows:

(5) BF=(BE−22.75−(5.45×BP))/8.95,

where, BF was the body fat (g/bird), BE was the body energy (kcal/bird), and BP was the body protein (g/bird). Then, the fat gain ratio was defined as:

(6) $$\mathrm{FGR}={\mathrm{BF}}_{f}-{\mathrm{BF}}_{i}.$$

where FGR was the fat gain ratio (g/bird in a period), and ${\mathrm{BF}}_{f}$ and ${\mathrm{BF}}_{i}$ the final and initial body fat (g/bird) contents in the period, respectively. Then, Equation (5) was inserted into Equation (6), replacing both ${\mathrm{BF}}_{f}$ and ${\mathrm{BF}}_{i}$, obtaining the following equation:

(7) $$\mathrm{FGR}=\left(\mathrm{EGR}-5.45\times \mathrm{PGR}\right)/8.95,$$

where EGR was the energy gain ratio (kcal/bird/d), and both FGR and PGR were expressed in g/bird/d. If not reported, the standard deviation (SD) was calculated as follows:

(8) $$\mathrm{SD}=\mathrm{SEM}\times \sqrt{n},$$

where SD was the standard deviation, SEM was the standard error of the mean, and n was the number of replications [22]. The SD of variables not reported in the primary studies (e.g., PFG and MKV) was calculated assuming that their coefficient of variation (CV; %) was the average of the CV values of those variables involved in their calculation and that:

(9) $$\mathrm{SD}=\left(\mathrm{CV}\times \mathrm{MV}\right)/100,$$

where SD was the standard deviation, CV was the coefficient of variation (%), and MV was the mean value of the response variable.

The grouping of the effect sizes per response variable was visually displayed as forest plots and their relationship with the changes in moderator variables as bubble plots (datapoints drawn as circles with sizes proportional to their precision).

### 2.6. Statistical Analyses

#### 2.6.1. Data Usefulness Determination: Basic Meta-Analyses (Random Models)

This study aimed to model changes in the effect sizes based on changes in moderator variables. Considering that the effect sizes resulted from applying multiple factors tested in the primary studies, overall effect sizes were reported only to inform the weighted mean effect size. The data was considered useful if heterogeneity, as an indicator of data diversity [23], was detected in the effect sizes across primary studies (Cochran Q test; *p* < 0.01 [24]). The heterogeneity was determined with the following random-effects model:

(10) $$y_{i}=µ+u_{i}+e_{i},$$

where $y_{i}$ was the observed effect size in the *i*-th data row (*i*: 1, … *k*) and also

(11) $$y_{i}=\theta_{i}+e_{i},$$

where µ was the mean true effect, $\theta_{i}$ was the unknown true effect in the *i*-th data row, $e_{i}$ was the intra-experimental sampling error in the *i*-th data row, $u_{i}$ was the inter-experimental deviation regarding the overall effect size in the *i*-th data row, *k* was the number of data rows, being that $u_{i}∼N\left(0,\tau^{2}\right)$ and $e_{i}∼N\left(0,v_{i}\right)$, and both independently, where *N* denoted the normal distribution of the random inter-experimental deviation (u) and the intra-experimental sampling error (e), $\tau^{2}$ was the variability among the true effects in the different data rows, so that τ was the standard deviation of the true effect sizes across rows (pure heterogeneity); and $v_{i}$ was the approximately known sampling variance of the estimated effect size in the *i*-th data row. A weighted least squares method was applied where the weighing criterion was the inverse of the variance [25], as follows:

(12) $${\bar{\theta }}_{w}=\sum w_{i}\theta_{i}/\sum w_{i},$$

where ${\bar{\theta }}_{w}$ was the true weighted mean effect size; $w_{i}$ was the weighing factor applied for the *i*-th data row, $\theta_{i}$ was the true effect size in the *i*-th data row. Then, ${\bar{\theta }}_{w}$ was the weighted average of the true effects ($\theta_{i}$) in the set of *k* data rows, with weights equal to the inverse of the corresponding variances, as follows:

(13) $$w_{i}=1/v_{i}.$$

The heterogeneity was estimated with the REML (Restricted Maximum Likelihood) method [26] and evaluated with the Chi-square test (heterogeneous if *p* < 0.05 [27]). The normal distribution of model residuals was evaluated both with the Shapiro-Wilk test (non-normal if *p* < 0.05 [28]) and the confidence to rely on the Central Limit Theorem based on the sample size [29]. The statistic *I*^2^, defined as the portion (%) of the total variability attributed to pure heterogeneity, was also determined [14].

#### 2.6.2. Modeling the Relationships between Variables: Meta-Regressions (Mixed Models)

Mixed-effects models were fitted to the data, considering the effect size was also a function of the level of a moderator variable (a co-variate), as follows:

(14) $$y_{i}=\beta_{0}+\beta_{j}x_{ij}+\cdots +\beta_{p}x_{kp}+u_{i}+e_{i},$$

where $y_{i}$ was the observed effect size in the *i*-th data row (*i*: 1,… *k*), and also

(15) $$y_{i}=\theta_{i}+e_{i},$$

where $\theta_{i}$ was the unknown true effect in the *i*-th data row, $e_{i}$ was the intra-experimental sampling error in the *i*-th data row, $\beta_{0}$ was the intercept, $\beta_{j}$ was the slope of the *j*-th moderator variable (*j*: 1,… *p*), $x_{ij}$ was the value of the *j*-th moderator variable in the *i*-th data row, $u_{i}$ was the inter-experimental random deviation regarding the overall effect size in the *i*-th data row, $e_{i}$ was the intra-experimental sampling error in the *i*-th data row, and *k* was the number of data rows. Furthermore, in this case $u_{i}∼N\left(0,\tau^{2}\right)$, $e_{i}∼N\left(0,v_{i}\right)$, and the mentioned weighted least squares method was applied. The estimate of the coefficient of the moderator variable ($\beta_{1}$) was tested with the Chi-square test. The normal distribution of residuals was tested as mentioned earlier. When residuals were approximately normal, but the *p*-value was <0.05, the data were re-analyzed to determine the probability associated with the slope of the moderator ($\beta_{1}$) but applying a permutation test with 10,000 iterations [30]. The PR^2^ statistic (pseudo-R^2^ [31]), defined as the amount of variability in the effect sizes among data lines explained by the model, was calculated as follows [32]:

(16) $${\mathrm{PR}}^{2}=({{\hat{\tau }}^{2}}_{RE}-{{\hat{\tau }}^{2}}_{ME})/({{\hat{\tau }}^{2}}_{RE}),$$

where ${\mathrm{PR}}^{2}$ was the pseudo-$R^{2}$ statistic, ${{\hat{\tau }}^{2}}_{RE}$ was the total heterogeneity estimated through the random-effects model, and ${{\hat{\tau }}^{2}}_{ME}$ was the residual heterogeneity estimated through the mixed-effects model. The 95% confidence intervals (95%CI) of moderator estimates were calculated.

All relationships were assessed considering simple linear meta-regressions and the models fully reported if the slopes were significant (*p* < 0.05) [27]. Complementarily, the type of genetic line (fast- or slow-growing birds) and an interaction term were also included as moderators for the sole purpose of conducting a parallelism test [33] and determining if the relationships between BWG and FCR and between FCR and PFG were equivalent in both types of birds. The relationships (slopes) were considered parallel (i.e., equivalent) if the interaction terms showed *p* values > 0.05 [33].

#### 2.6.3. Bias Assessment

The risk of bias due to missing results was assessed combining the application of [30]: (1) Funnel plot-associated Trim-and-Fill method to identify a possible number of missing values and obtain an estimated set of them. (2) Egger’s test to determine the existence of bias (bias if *p* < 0.05) in the dataset. (3) Comparing the 95%CI of the overall effect sizes calculated with the original and adjusted datasets (including the Trim-and-Fill obtained values) to determine the existence of bias in the overall effect size. The overall effect size was considered biased if the following two criteria were met: (A) Estimates were statistically different (*p* < 0.05; both overall effect sizes fell outside the 95%CI of the counterpart [34]) and (B) the boundaries of the 95%CI had the same sign (positive or negative). Biases were considered influential if inferences were built based on the overall effect sizes.

### 2.7. Software

The data was analyzed using R programming language [35] with RStudio 1.1.456 interface [36]. The goodness-of-fit to the normal distribution was tested using the package *stats* [35], the meta-analysis procedures, including the Egger’s asymmetry test with *Metafor* 2.0-0 [25], and the graphs plotting with *lattice* 0.20-38 [35].

## 3. Results

### 3.1. Study Selection

**Figure 1:**
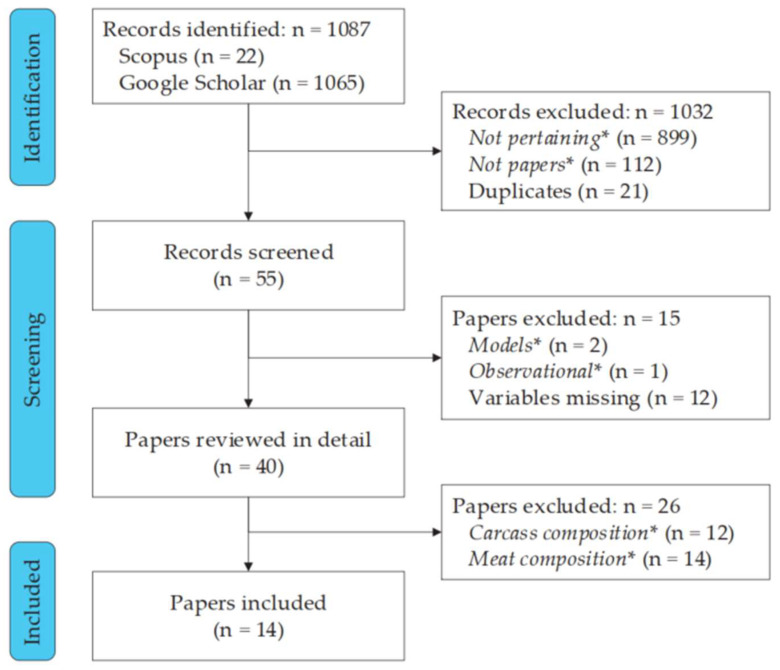
PRISMA flowchart of the study search and selection process. * *Not pertaining*, documents not related to the focus of the study; *Not papers*, documents not actual papers published in scientific journals; *Models*, papers reporting mathematical models, but not mean values per treatment; *Observational*, observational studies; *Carcass composition* or *Meat composition*, chemical composition not determined in the whole bird but the carcass or meat, respectively.

Searches conducted provided 1087 records, from which 1032 were removed due to not pertaining to the focus of the study (899), through title inspection, not being scientific papers (112), or because of duplicates (21). Fifty-five papers were screened, from which the following were excluded: two papers presenting models but not treatment mean and variability values, one observational study, and 12 not reporting results in the three target groups of response variables. After reviewing in detail 40 papers, 26 were excluded because the chemical composition was determined for the carcass (12 papers) or meat (14 papers) and did not determine the chemical composition of the whole bird (Figure 1). The remaining 14 papers were included in the study (Table 1).

### 3.2. Characteristics of Studies Included

A widely diverse database was built, including 78 data rows from the 14 scientific papers published between 1992 and 2019 in nine scientific journals. Fifteen rows included females and 57 male birds. Both fast-(two genetic lines; sampled between 21 and 49 days of age; 31 rows) and slow-(four genetic lines; sampled between 21 and 105 days of age; 47 rows) growing birds were included. The database included the following factors tested: Ambient temperature (9 rows), number of feeding phases (2 rows), feed restriction (1 row), source of dietary amino acids (intact protein or industrial amino acids; 6 rows), dietary methionine precursors (10 rows), organic trace minerals (8 rows), and dietary contents of energy (17 rows), protein (7 rows), and amino acids (18 rows).

The database included 3744 protein or N analyses to determine protein gain, 2336 analyses of fat or gross energy in bomb calorimeters to determine the fat gain, 1896 birds used to determine processing traits, and 1000 independent groups including a total of 22,256 birds to determine performance.

### 3.3. Data Usefulness

All assessed variables showed heterogeneity among rows (Table 2). The tau (τ) values found indicated that the treatments tested in the primary studies, whatever they were, produced effects for BWG, PGR, and FGR that typically varied from −8.4 to +8.4, −1.9 to +1.9, and −0.5 to +1.3 g/bird/d, respectively. The effects on PFG varied from −0.35 to +0.20, on FCR from −42 to +28 points, on CAR from −0.60 to +0.97 percent points, on BLR from −0.04 to +0.03, and on MKV from −0.14 to +0.01 $/bird. Consequently, the data in all tested variables were considered useful. Detailed effect sizes and their corresponding 95%CI are presented in Figures S1–S5. The BLR and MKV datasets and the BLR weighted mean effect sizes in the BLR showed bias (Table S1). These did not influence the data’s usefulness as the overall effect size was relevant only to report the weighted mean effect size, based on the general approach used in the study. Therefore, they were not considered influential.

### 3.4. Relationships between Variables

**Figure 2:**
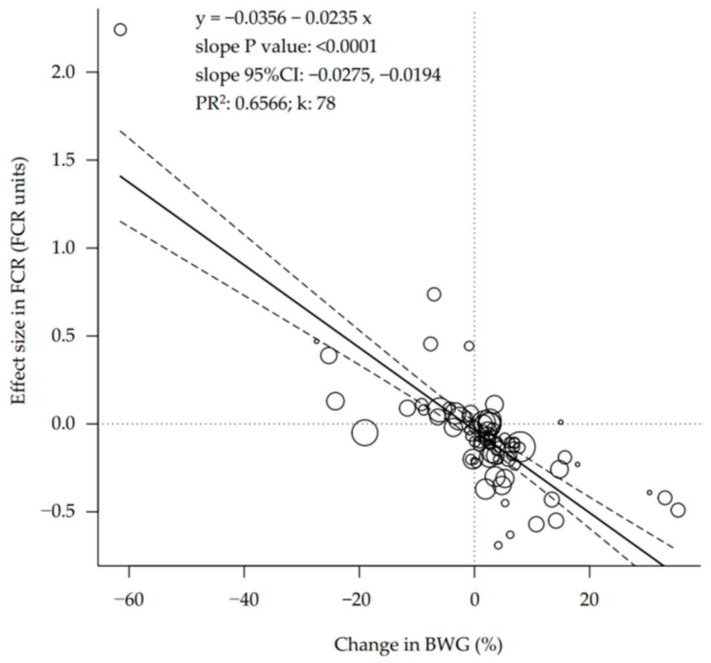
Bubble plot of the relationship between growth rate and feed efficiency of broilers. The feed conversion ratio (FCR) changes are associated with changes in body weight gain (BWG). 95%CI = 95% confidence interval. PR^2^ = pseudo R^2^. k = number of studies or data rows. Each circle is a primary study or data row, and its size is proportional to the relative weight given to calculate the regression, based on its precision. The continuous straight line is the prediction line, and the dashed curves are the boundaries of its 95% confidence interval.

Several relationships were explored between performance, body composition, and processing yield variables. The changes in BWG were related to the effect sizes in FCR (Figure 2; *p* < 0.001), and every percentage point increase in the BWG was associated with a reduction in FCR of 23.5 g less feed (95%CI: 19.4 to 27.5) per kg of body weight gain. This relationship was equivalent in slow- and fast-growing birds (parallel slopes; interaction *p* = 0.602; Table 3).

**Figure 3:**
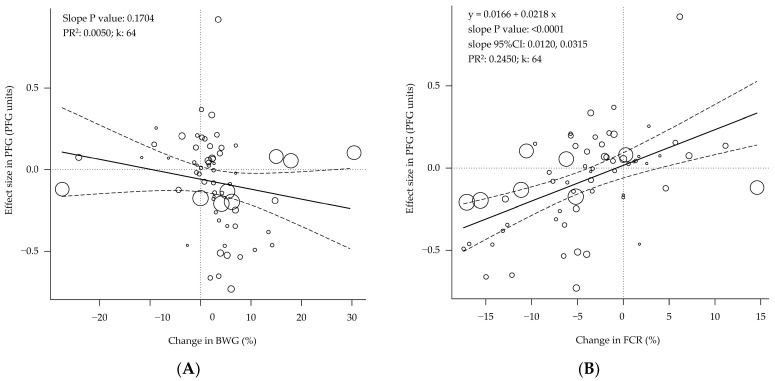
Bubble plot of the relationship between performance and body composition of broilers. The changes in body weight gain (**A**) are not associated with changes in body composition, but the changes in feed conversion ratio (**B**) are associated with. 95%CI = 95% confidence interval. PR^2^ = pseudo R^2^. k = number of studies or data rows. Each circle is a primary study or data row, and its size is proportional to the relative weight given to calculate the regression, based on its precision. The continuous straight lines are the prediction lines, and the dashed curves are the boundaries of their 95% confidence intervals.

The changes in BWG were not shown to be related to the effect sizes in PFG (Figure 3A; *p* < 0.170), but those changes in the FCR were (Figure 3B; *p* < 0.001), so the reduction in FCR was associated with reductions in the PFG. This relationship was consistent in slow- and fast-growing birds (parallel slopes; interaction *p* = 0.900; Table 3).

**Figure 4:**
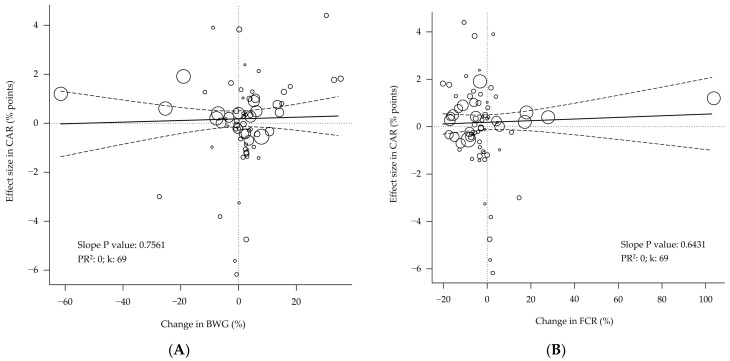

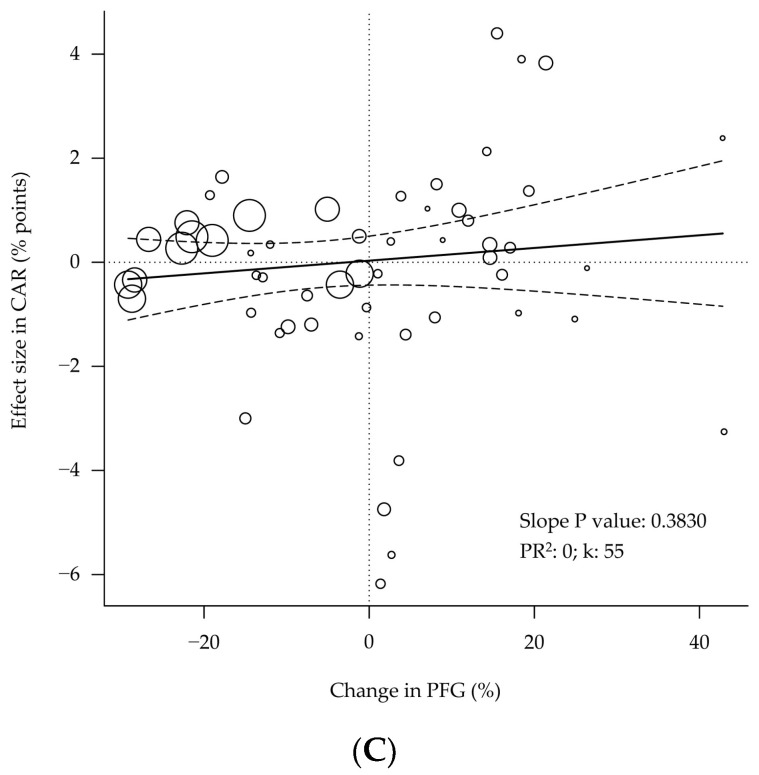
Bubble plot of the relationship between performance and body composition with carcass yield of broilers. The changes in the body weight gain (**A**), feed conversion ratio (**B**), or protein-to-fat gain ratio (**C**) show no association with the changes in carcass yield. PR^2^ = pseudo R^2^. k = number of studies or data rows. Each circle is a primary study or data row, and its size is proportional to the relative weight given to calculate the regression, based on its precision. The continuous straight lines are the prediction lines, and the dashed curves are the boundaries of their 95% confidence intervals.

**Figure 5:**
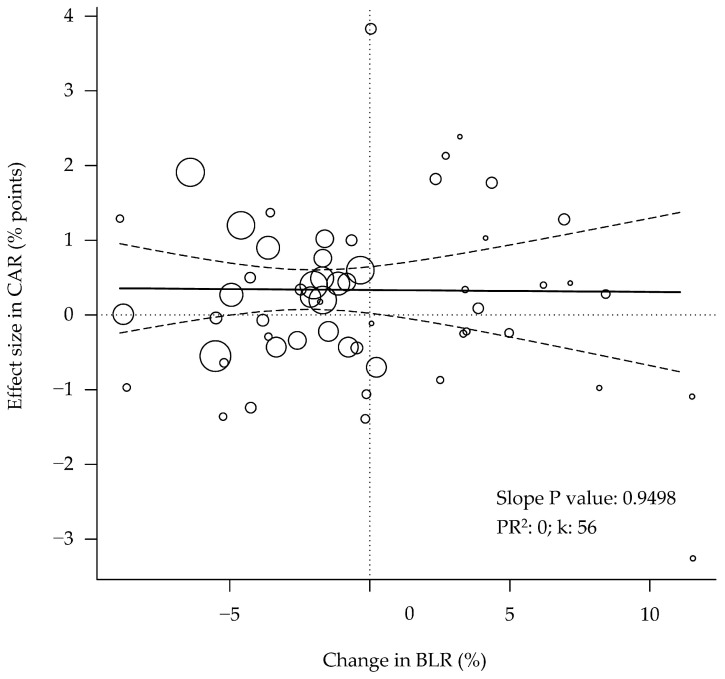
Bubble plot of the changes in carcass yield and those in carcass conformation, as measured by the breast-to-legs ratio, are not related. 95%CI = 95% confidence interval. PR^2^ = pseudo R^2^. k = number of studies or data rows. Each circle is a primary study or data row, and its size is proportional to the relative weight given to calculate the regression, based on its precision. The continuous straight line is the prediction line, and the dashed curves are the 95% confidence interval boundaries.

The effect sizes in CAR showed no relationship with the changes in BWG, FCR, or PFG (Figure 4). In addition, the carcass yield showed no relationship with the carcass conformation, as measured by the BLR (Figure 5; *p* = 0.950).

**Figure 6:**
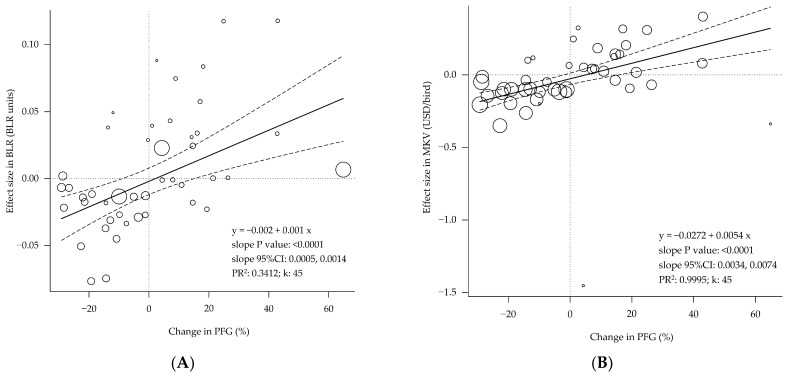
Bubble plot of the changes in body composition are associated with the changes in carcass conformation (**A**) and market value (**B**) of the broilers. 95%CI = 95% confidence interval. PR^2^ = pseudo R^2^. k = number of studies or data rows. Each circle is a primary study or data row, and its size is proportional to the relative weight given to calculate the regression, based on its precision. The continuous straight lines are the prediction lines, and the dashed curves are the boundaries of their 95% confidence intervals.

The data showed that the changes in the body composition were highly related to those in the carcass conformation (i.e., breast-to-legs ratio) and the carcass market value (considering the breast and legs market prices; Figure 6). Every percent point increase in the PFG translates into a 0.001 increase in the BLR and represents 0.54 cents marginal value increase per carcass.

## 4. Discussion

The relationships between the changes in performance, body composition, and processing yield were evaluated. A database with 78 data rows was extracted from 14 primary studies testing multiple interventions (88% data rows related to nutrition). Broiler response was determined for the variables (performance, body composition, and processing yield) in the database.

In the present study, the heterogeneity of data was considered a formal verification of its usefulness to explore the influence of moderator variables on the effect sizes. Indeed, not only was the FCR (effect size SD = 35 FCR points), but most of the data used were highly diverse and heterogeneous (Table 2). The wide range of values in the tested variables increased the ability to build robust inferences from the models, so predictions are more likely to be interpolations. This can be considered a strength of the models in this study. Making statements outside the range of data used to develop the models (i.e., extrapolation) does not guarantee their accuracy and precision, reducing their validity [34].

The data demonstrates that regardless of the intervention applied to improve the performance, the higher the BWG, the better the feed efficiency (Figure 2). This finding demonstrates how important it is to ensure the broilers grow fast to support the sustainability of the poultry industry. The reduction of natural resources used (i.e., feed usage) as the FCR improves ultimately reduces the carbon footprint per kg of meat produced [2]. This consideration and the fact that both slow- and fast-growing broilers show the same relationship between BWG and FCR imply that producing slow-growing birds may not be sustainable. Furthermore, these operations need to ensure their birds grow as fast as possible to reduce their FCR and support their profitability. Indeed, a higher water footprint (L per kg of meat produced) for slow-growing birds can be inferred from the literature based on an equivalent water consumption per unit of weight gain [51] but a lower breast yield [52]. Finally, it may be contradictory to slow down the growth rate of the broilers to achieve a claimed better welfare (e.g., less skeletal integrity issues [53]) and then have to try to speed it up.

Interestingly, the data showed that the better the FCR, the lower the PFG (Figure 3), both in fast- and slow-growing birds (Table 3). This relationship does not imply that the better the FCR, the fatter the bird. It indicates that when the FCR improves as the result of a treatment, the fat gain (g/day) increases in a higher percentage than the protein gain (g/day). This relationship is consistent with the higher energy efficiency for fat gain versus protein gain [2]. The lower energy efficiency for protein gain has been associated with a higher heat production per unit of protein gain than fat gain in swine [54] and a higher fasting heat production per unit of actual body protein mass than fat mass in broilers due to protein turnover [5]. Furthermore, considering that the whole body fat content is lower than the protein content [29,55], an increased percentage of BWG from fat gain compared to protein gain is more likely to decrease the PFG.

The analyses showed that the effect sizes observed in the carcass yield are unrelated to the changes in performance, body composition (Figure 4), or even carcass conformation (Figure 5). The absence of a relationship between the carcass yield and the PFG in the study may be explained by the low variability in the carcass yield (effect size SD < 1 percent point) and the lower number of data rows available (k = 55; 30% lower sample size [56,57]. The carcass yield depends mainly on the relative weights of the carcass, viscera, and abdominal fat [53]. A higher PFG is expected to be associated with a lower abdominal fat content. It has been reported that the yield of the whole carcass is hardly affected by dietary factors [58]. However, the breast yield seems to be more sensitive to nutritional interventions [58] than the whole carcass yield. Indeed, it has been reported that the dietary supplementation of methionine produced effects (*p* < 0.05) that were more prominent in the abdominal fat yield (−13.8%) and the breast meat yield (+6.9%) than in the whole carcass yield (+0.8%) [59].

Finally, the observed association of the changes in body composition, as measured by the PFG, with the effect sizes in the carcass conformation agree with the higher protein content in the breast (64.6%) compared to the legs (50.8%) and the opposite relationship in the fat content (breast, 24.7%; legs, 31.7%) [37]. This condition explains the tight relationship between the body composition (PFG) and the marginal extra value of the carcass (MKV; Figure 6). As an example, a 40% change in the PFG (the difference between the mean positive and negative changes in the present study; +20% versus −20%) would represent a difference of $0.216 per bird, equivalent to $22 million of extra value in a broiler operation producing 100 million broilers/year. This finding supports that body composition may be used as a predictor of the economic value of broiler production.

Future research may explore the dynamics and relationships between the body protein and fat content and their ratio. The findings in the present study will support better modeling approaches to predict body energy retention, as calculated from the protein and fat gains and their corresponding caloric coefficients, and ultimately favor the determination of the net productive energy (Arkansas Net Energy). In addition, practical body composition measurements, such as the abdominal fat pad weight [60], may be explored as indicators of the final carcass conformation.

### Limitations and Interpretation

Even though all the data utilized in the meta-regression analyses were from actual experiments, our findings do not establish formal cause-effect relationships but associations.

All data available fitting the required criteria were included in this study; however, the factors tested in the primary studies were mainly focused on nutrition. Furthermore, none of the studies included specific factors that may influence the relationships established herein, such as stress [61], gut microbiota changes [62], and coccidia, *Clostridium perfringens*, or feed ingredient-associated specific enteric challenges [18]. Therefore, the relationships established herein may change with new data.

One of the biggest challenges in mathematically modeling biological systems is that researchers need to balance three opposing criteria (precision, generality, and realism), where only two of them can be optimized at a time (the Levins’ Three Dimensions framework [63]). Usually, research in animal and poultry sciences is conducted under well-controlled conditions, favoring precision and realism at the expense of generality. Then, results differ when research conditions change (e.g., commercial conditions). In contrast, the general approach of the present study prioritized generalization and realism at the expense of precision [63]. The dataset included studies with extremely diverse characteristics, such as the year of publication (up to 30 years back), treatment factors tested, ages, genetics, dietary energy and amino acid contents, and both sexes. Even though these factors introduced variability in the data, some relationships were successfully characterized. Furthermore, those uncontrolled factors were random ones. Consequently, the relationships established in the present study are more generalizable than inferences from a single study under certain conditions. The influence of the varying conditions was intentionally not assessed to avoid misleading inferences based on insufficient statistical power. However, all effect sizes came from factors tested within the same genetic line; therefore, the generalizations in our study do not include relationships among genetic lines [64,65,66].

In addition, it should be considered that the actual performance, body composition, and carcass values were not used in the study but the changes that resulted from experimental treatments. Therefore, the influence of uncontrolled factors was minimized. This way, the differences in performance and body composition between sexes or across the years did not influence the calculations, but their relative sensitivity did. For example, the data showed that the relationship between the changes in body weight gain and feed conversion ratio is virtually the same in fast- and slow-growing birds, even though their genetics differ. Even though performance, lean mass, and breast meat yield have evolved with genetics across the years, it is unlikely that the relationship between the percent changes in body composition and the percent changes in processing yield as the result of an experimental treatment has done so.

Finally, the database available from the literature will increase if only two of the targeted areas are included (broiler performance, body composition, and processing yield) instead of including all three. If, for example, the same procedure is replicated but restricting the searches only to performance and body composition, more data will be found, and the influence of the genetics (i.e., year of publication), sex, and nutrient profile of the diets may be assessed.

## 5. Conclusions

In conclusion, the results indicate that positive changes in body weight gain are associated with improvements in the feed conversion ratio and that the body composition predicts the carcass conformation and its market value. The results of the study on the association between body weight gain and the feed conversion ratio imply the importance of maximizing broiler weight gain to support the sustainability of broiler production.

## Figures and Tables

**Table 1:** Characteristics of the studies used to determine the relationships between performance, body composition gain, and processing yield in broilers.

Study	Genetic	Type ^1^	Sex ^2^	Factors Tested	Age, d	Data Rows
Faria Filho et al., 2005 [37]	Cobb	FG	M	Ambient temperature	7–21	4
				Dietary protein content		
Faria Filho et al., 2006 [38]	Cobb	FG	M	Ambient temperature	43–49	4
				Dietary protein content		
Hauschild et al., 2015 [39]	Cobb	FG	M, F	Number of feeding phases	1–42	2
Liu et al., 2019 [40]	Huaixiang	SG	M	Ambien temperature	36–105	4
Mendonca et al., 2007 [41]	ISA Label	SG	F	Dietary energy content	22–85	8
Mendonca et al., 2008 [42]	ISA Label	SG	M	Dietary energy content	22–70	8
Nascimento et al., 2009a [43]	ISA Label	SG	M, F	Dietary amino acids content	57–84	6
Nascimento et al., 2009b [44]	ISA Label	SG	M, F	Dietary amino acids content	57–84	6
Oliveira et al., 2014 [45]	Cobb	FG	M	Source of dietary amino acids	22–42	6
Perrault and Leeson, 1992 [46]	NA	FG	M	Ambient temperature	1–35	3
				Dietary energy content		
				Feed restriction		
Trindade Neto et al., 2009 [47]	Ross	FG	M	Dietary amino acids content	36–49	4
Trindade Neto et al., 2010 [48]	Ross	FG	M	Organic trace minerals*	22–42	8
Xi et al., 2007 [49]	LinNan	SG	M	Dietary protein content	1–63	9
				Methionine precursor		
Yao et al., 2006 [50]	Avian	SG	NA	Methionine precursor	8–42	6

^1^ FG, fast-growing; SL, slow-growing. ^2^ M, males; F, females; NA, not available.

**Table 2:** Heterogeneity in the effect sizes in each variable assessed in the study.

Variables	Effect Sizes ^1^		Heterogeneity ^2^		
	Range	Weighed Mean	τ	*p*-Value	$I^{2}$
Body weight gain (g/bird/d)	−58.0 to +20.5	−0.0003	8.4118	<0.001	98.94
Feed conversion ratio	−0.69 to +2.24	−0.0690	0.3483	<0.001	98.30
Protein gain ratio (g/bird/d)	−16.61 to +2.21	−0.0039	1.8829	<0.001	97.40
Fat gain ratio (g/bird/d)	−3.29 to +2.50	0.4348	0.8846	<0.001	88.19
Protein-to-fat gain ratio	−0.73 to +0.92	−0.0744	0.2725	<0.001	94.22
Carcass yield (%)	−6.18 to +4.40	0.1867	0.7851	<0.001	47.45
Breast-to-legs ratio	−0.08 to +0.12	−0.0072	0.0334	<0.001	82.67
Market value ($/bird)	−1.45 to +0.40	−0.0626	0.0736	0.016	28.47

^1^ Expressed in the same units as the variable. ^2^ τ is the standard deviation of the true effect sizes across primary studies (pure heterogeneity) expressed in the units of the corresponding variable. *I*^2^ is the portion (%) of the total variability attributed to pure heterogeneity among studies.

**Table 3:** Slow- and fast-growing genetic lines show equivalent relationships between the changes in growth rate and feed efficiency and between those in feed efficiency and body composition ^1^.

Parameters	Estimate	*p* Value *	PR^2^	k
Effect size in FCR				
Intercept	0.0390	0.526	0.6830	78
Change in BWG, %	−0.0230	0.001		
Slow growing type	−0.1371	0.003		
Interaction	0.0024	0.602		
Effect size in PFG				
Intercept	0.1449	0.066	0.3166	64
Change in FCR, %	0.0192	0.098		
Slow growing type	−0.2005	0.015		
Interaction	−0.0016	0.900		

^1^ FCR, feed conversion ratio; BWG, body weight gain; PFG, protein-to-fat gain ratio. PR^2^, pseudo-R^2^ statistic. k, number of studies or data rows. * *p* values determined by a permutation test. *p* values > 0.05 denote a lack of interaction, parallel slopes, and equivalent relationships between primary predictors (change in BWG; change in FCR) and response variables (effect sizes in FCR and PFG).

## Data Availability

Not applicable.

## Supplementary Materials

The following supporting information can be downloaded at: https://www.mdpi.com/article/10.3390/ani12192706/s1, Figure S1: Effect sizes on the FCR; Figure S2: Effect sizes on the PFG; Figure S3: Effect sizes on the CAR; Figure S4: Effect sizes on the BLR; Figure S5: Effect sizes on the MKV; Table S1: Assessment of bias.
