# Supplementary material for: The Relationship between Performance, Body Composition, and Processing Yield in Broilers: A Systematic Review and Meta-Regression
Source: Animals (Basel). 2022 Oct 8;12(19):2706. doi: 10.3390/ani12192706 (PMC9559297; doi:10.3390/ani12192706)
Supplement: Supplementary file 1 [file animals-12-02706-s001.zip › Figure S5 - Effect sizes on the MKV.pdf]

EFFECT SIZES ON MARKET VALUE

Black squares are the effect sizes. The size of the squares denote the relative weight given to that specific effect size.
The horizontal lines crossing the effect sizes represent the associated 95% confidence intervals.
The black diamond is the weighed mean effect size and the associated dotted line represents its 95% confidence interval.

Reference	Relative weight	Effect [95% CI]
Faria Filho et al. 2005	2.95%	−0.19 [−0.35, −0.03]
Faria Filho et al. 2005	3.17%	−0.21 [−0.36, −0.06]
Faria Filho et al. 2005	3.41%	−0.06 [−0.20, 0.08]
Faria Filho et al. 2005	3.51%	−0.25 [−0.38, −0.11]
Faria Filho et al. 2006	3.13%	−0.07 [−0.22, 0.08]
Faria Filho et al. 2006	3.12%	−0.05 [−0.20, 0.10]
Faria Filho et al. 2006	3.12%	−0.02 [−0.17, 0.13]
Faria Filho et al. 2006	3.13%	−0.07 [−0.22, 0.08]
Hauschild et al. 2015	0.26%	0.12 [−0.59, 0.83]
Hauschild et al. 2015	0.26%	0.32 [−0.38, 1.03]
Mendonca et al. 2007	2.80%	−0.12 [−0.28, 0.05]
Mendonca et al. 2007	2.79%	−0.10 [−0.27, 0.07]
Mendonca et al. 2007	2.78%	−0.05 [−0.22, 0.12]
Mendonca et al. 2007	2.82%	−0.21 [−0.37, −0.04]
Mendonca et al. 2007	2.40%	−0.10 [−0.29, 0.09]
Mendonca et al. 2007	2.40%	−0.10 [−0.29, 0.09]
Mendonca et al. 2007	2.40%	−0.10 [−0.29, 0.09]
Mendonca et al. 2007	2.46%	−0.35 [−0.54, −0.16]
Mendonca et al. 2008	2.20%	−0.17 [−0.37, 0.03]
Mendonca et al. 2008	2.18%	−0.09 [−0.30, 0.11]
Mendonca et al. 2008	2.22%	−0.26 [−0.46, −0.06]
Mendonca et al. 2008	2.20%	−0.20 [−0.40, 0.01]
Mendonca et al. 2008	2.21%	−0.10 [−0.30, 0.10]
Mendonca et al. 2008	2.22%	−0.13 [−0.33, 0.08]
Mendonca et al. 2008	2.22%	−0.14 [−0.35, 0.06]
Mendonca et al. 2008	2.19%	−0.01 [−0.22, 0.19]
Nascimento et al. 2009a	1.16%	0.05 [−0.26, 0.36]
Nascimento et al. 2009a	1.20%	−0.09 [−0.40, 0.21]
Nascimento et al. 2009a	1.19%	−0.05 [−0.36, 0.26]
Nascimento et al. 2009a	1.09%	0.04 [−0.28, 0.37]
Nascimento et al. 2009a	1.03%	0.32 [−0.02, 0.65]
Nascimento et al. 2009a	1.07%	0.14 [−0.19, 0.47]
Nascimento et al. 2009b	1.70%	0.14 [−0.10, 0.39]
Nascimento et al. 2009b	1.78%	−0.12 [−0.36, 0.12]
Nascimento et al. 2009b	1.78%	−0.12 [−0.35, 0.12]
Nascimento et al. 2009b	1.57%	−0.04 [−0.29, 0.22]
Nascimento et al. 2009b	1.56%	0.02 [−0.24, 0.28]
Nascimento et al. 2009b	1.56%	0.03 [−0.23, 0.28]
Oliveira et al 2014	0.78%	0.16 [−0.24, 0.55]
Oliveira et al 2014	0.77%	0.24 [−0.15, 0.64]
Oliveira et al 2014	0.78%	0.20 [−0.19, 0.60]
Oliveira et al 2014	0.92%	−0.24 [−0.59, 0.12]
Oliveira et al 2014	0.91%	−0.17 [−0.53, 0.19]
Oliveira et al 2014	0.91%	−0.13 [−0.49, 0.23]
Perrault and Leeson 1992	0.06%	−0.20 [−1.71, 1.32]
Perrault and Leeson 1992	0.06%	−0.34 [−1.85, 1.18]
Perrault and Leeson 1992	0.06%	−1.45 [−2.97, 0.07]
Trindade Neto et al 2009	0.62%	0.10 [−0.35, 0.55]
Trindade Neto et al 2009	0.61%	0.25 [−0.20, 0.70]
Trindade Neto et al 2009	0.62%	0.06 [−0.38, 0.51]
Trindade Neto et al 2009	0.62%	0.13 [−0.32, 0.57]
Trindade Neto et al 2010	1.39%	−0.04 [−0.32, 0.24]
Trindade Neto et al 2010	1.31%	0.31 [0.02, 0.60]
Trindade Neto et al 2010	1.29%	0.40 [0.11, 0.69]
Trindade Neto et al 2010	1.34%	0.20 [−0.08, 0.49]
Trindade Neto et al 2010	1.45%	−0.07 [−0.34, 0.20]
Trindade Neto et al 2010	1.39%	0.18 [−0.09, 0.46]
Trindade Neto et al 2010	1.42%	0.04 [−0.23, 0.32]
Trindade Neto et al 2010	1.41%	0.08 [−0.20, 0.36]
RE Model	100.00%	−0.06 [−0.10, −0.03]
95% prediction interval		[−0.21, 0.09]

Effect, P = 0.0009 / Heterogeneity, P = 0.0156

Effect sizes (USD/bird)

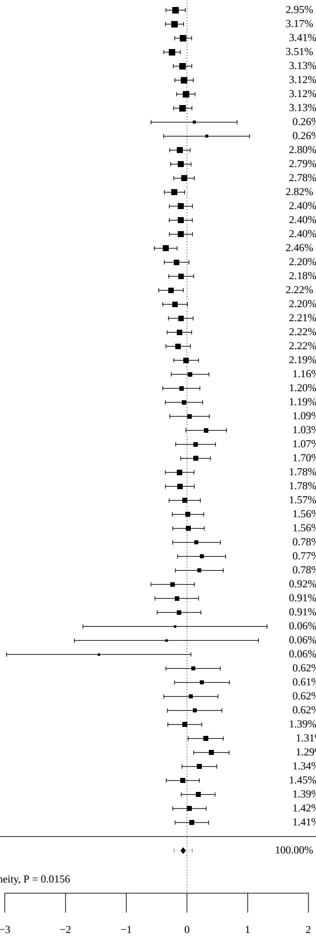

Effect size (USD/bird)
