# Supplementary material for: The Relationship between Performance, Body Composition, and Processing Yield in Broilers: A Systematic Review and Meta-Regression
Source: Animals (Basel). 2022 Oct 8;12(19):2706. doi: 10.3390/ani12192706 (PMC9559297; doi:10.3390/ani12192706)
Supplement: Supplementary file 1 [file animals-12-02706-s001.zip › Table S1 - Assessment of bias.pdf]

Table S1. Assessment of bias in the data used and in the weighed mean effect sizes.

Response variable [1]	Bias in the dataset		Bias in the weighed mean effect size	
	Trim-and-Fill [2]	Egger's test P value [3]	Estimate and 95%CI [4]	Inference [5]
FCR	28	0.7065	O: -0.0690 (-0.1483; +0.0103)<br>A: +0.0681 (-0.0065; +0.1427)	NEB
PFG	0	0.7144	O: -0.0744 (-0.1479; -0.0010)<br>A: -0.0744 (-0.1479; -0.0010)	NEB
CAR	10	0.1036	O: +0.1867 (-0.1261; +0.4996)<br>A: +0.4946 (+0.0861; +0.9030)	NEB
BLR	15	<0.0001	O: -0.0072 (-0.0174; +0.0029)<br>A: -0.0216 (-0.0335; -0.0096)	B
MKV	14	0.0005	O: -0.0626 (-0.0996; -0.0255)<br>A: -0.1050 (-0.1487; -0.0613)	NEB

[1] FCR, feed conversion ratio; PFG, protein-to-fat gain ratio; CAR, carcass yield (%); BLR, breast-to-leg quarters ratio; MKV, market value ($/bird).

[2] Estimated number of missing data rows.

[3] Bias if $P < 0.05$.

[4] 95%CI, 95% confidence interval; O, original dataset; A, dataset including the set of missing values estimated with the Trim-and-Fill method.

[5] NEB, no evidence of bias; B, biased.
